# Psychotropic medication non-adherence and associated factors among adult patients with major psychiatric disorders: a protocol for a systematic review

**DOI:** 10.1186/s13643-018-0676-y

**Published:** 2018-01-22

**Authors:** Agumasie Semahegn, Kwasi Torpey, Abubakar Manu, Nega Assefa, Gezahegn Tesfaye, Augustine Ankomah

**Affiliations:** 1 Department of Population, Family and Reproductive Health, School of Public Health, College of Health Sciences, University of Ghana, Legon, Accra, Ghana; 2 College of Health and Medical Sciences, Haramaya University, Po. Box 235, Harar, Ethiopia

**Keywords:** Medication non-adherence, Psychiatric disorders, Systematic review, Meta-analysis

## Abstract

**Background:**

Evidence from the global burden of diseases show that psychiatric disorders are a growing public health concern. Maintaining adherence to medication is the most essential, but challenging course in the pharmacological treatment modality for major psychiatric disorders. Nevertheless, there is a paucity of abridged evidence on the level of psychotropic medication non-adherence and associated factors. Therefore, we aim to systematically summarize existing primary studies finding to estimate the level and identify associated factors of psychotropic medication non-adherence among adult patients with major psychiatric disorders.

**Methods:**

We will search studies using computerized search engines, main electronic databases and other relevant sources. PubMed (Medline), EMBASE, CINAHL, PsycINFO, Web of Science, WHO Global Health Library, and direct Google search will be searched to retrieve studies written in English language before December 2017. Observational studies (cross-sectional, case-control, cohort or longitudinal, survey and surveillance reports) on major psychiatric disorders (schizophrenia, major depressive and bipolar disorders) among adult patients will be eligible. Data will be extracted independently by two authors. Data synthesis and statistical analysis will be carried out. Pooled estimate will be done to quantify the level of psychotropic medication non-adherence using Comprehensive Meta-Analysis software.

**Discussion:**

Psychiatric disorders remain a public health, social and economic concern worldwide. Management of major psychiatric disorders is highly affected by medication non-adherence. Thus, undertaking an integrated and multifaceted approach is necessary to reduce the burden of medication non-adherence, and enhance the quality of patients’ life. Evidence is required to design appropriate intervention to prevent psychotropic medication non-adherence.

**Protocol registration:**

PROSPERO: 2017: CRD42017067436.

**Electronic supplementary material:**

The online version of this article (10.1186/s13643-018-0676-y) contains supplementary material, which is available to authorized users.

## Background

Globally, psychiatric disorders has been a public health challenge, and attributed 14% to the global burden of diseases. Approximately 450 million people have been affected by psychiatric disorders [1–4]. Depressive disorders (350 million), bipolar disorders (60 million), and schizophrenia (21 million) are the most common psychiatric conditions [1]. Psychiatric disorders are the main cause (31.7%) of the years lived with long term disabilities and dependency. Of these, unipolar depression (11.8%), schizophrenia (2.8%) and bipolar disorder (2.4%) have been contributing for the disabilities [5]. Psychiatric disorders have worsened as a result of ineffective treatments, coverage, and poor adherence. The cost has estimated to be approximately US$2.5 trillion in 2010, and is expected to rise up to US$6.0 trillion by 2030. Misplaced resources, vanished production, unemployment, absence from work, and premature mortality are some of the indirectly incurred economic costs due to medication non-adherence [6].

A person with major psychiatric disorders (depression and schizophrenia) has a 40–60% greater chance of dying prematurely than the general population. Notwithstanding, the health system has not adequately and effectively responded to psychiatric disorders. Poor quality of care for those patients who are receiving treatment still persists. However, the World Health Organization (WHO) has designed comprehensive strategic action plan (2013–2020) to promote mental well-being, prevent psychiatric disorders, provide care, enhance recovery, and reduce morbidity, disability, and mortality [7]. Most psychiatric disorders are common in people who are living alone, have poor physical health, and are unemployed [8]. Psychiatric disorders are associated with individual and families’ living standard, socio-economic, cultural, community social support, political, and environmental factors [7].

Maintaining adherence to the medication is the crucial, and also a challenging course in the pharmacological management of major psychiatric disorders [3, 9–11]. According to the WHO, medication non-adherence is defined as “a case in which a person’s behavior in taking medication does not correspond with agreed recommendations from health personnel”. It can be either intentional or unintentional, including failing to initially fill or refill a prescription, discontinuing a medication before completing the course of therapy, taking more or less of a medication than prescribed and taking a dose at the wrong time [12]. Patients who are suffering from major psychiatric disorders are most likely to be non-adherent to their medication [13, 14]. Understandably, major psychiatric disorders have an effect on patients’ reasoning skills and insight which can negatively affect adherence [11].

Patients with depressive symptoms had not taken almost half of the prescribed doses within 3 months of initiation of the therapy. The rates of adherence are low at 50–60% and 35% for schizophrenia and bipolar affective disorders, respectively [3]. Furthermore, patients with major psychiatric disorders with medication non-adherence can cause exacerbation of their illness and complications which lead to re-hospitalization, poor psychosocial outcomes, relapse of symptoms, reduce effectiveness of subsequent treatment, wastage of limited health care resources, increase substance abuse, poor quality of life, and increased suicide [3, 10, 11, 15–18].

Research evidence on psychotropic medication non-adherence and associated factors of major psychiatric disorders patients is essential to design appropriate intervention, and achieve desired treatment goal for both patients and health care providers. Nevertheless, there is a paucity and growing demand of systematic review and meta-analysis on the level of medication non-adherence and associated factors. However, many primary studies have been done and published on this issue. Therefore, the aim of this systematic review and meta-analysis is to summarize available primary studies finding to determine level of psychotropic medication non-adherence and associated factors.

## Methods

### Protocol development and registration

This systematic review and meta-analysis protocol has been registered in the International Prospective Register of Systematic Reviews (PROSPERO) (ID: CRD42017067436). This protocol is developed, and then report will be compiled according to the recommendation of the Preferred Reporting Items for Systematic Review and Meta-Analysis Protocols (PRISMA-P) 2015 [19] (Additional file 1).

### Search methods for identification of studies

We will search studies using electronic search, hand search, and email. Search strings will be constructed based on the research question. Then, studies will be searched from the main electronic databases (PubMed (Medline), EMBASE, CINAHL, PsycINFO and Web of Science), WHO global health library, direct Google search, and other relevant sources before December, 2017. In addition, emails will be sent to authors whose studies will be included in the systematic review, and if their email is available to request publications they might have. For those who might not respond to the email request on time, a follow up email will be re-sent at most three times, every 7 days for their kind response. In addition, relevant citations from retrieved studies will be searched. Search strings are constructed using keywords and their combinations (Additional file 2). However, search strings will be modified to suit to the databases interface accordingly. The overall identified studies will be exported to the EndNote citation manager [20], and duplicates will be excluded.

### Eligibility criteria

Observational studies (cross-sectional, case-control, cohort or longitudinal, survey, and surveillance) will be included in the selection process. In addition, studies that have been done among adult patients (aged 18 years and older) with major psychiatric disorders (schizophrenia, major depressive, and bipolar disorders) will be eligible for the systematic review. All studies (published or unpublished) that have been written in English language will be retrieved and included into reviewed process. Nevertheless, all studies that considered psychiatric disorders as a factor for other (non-psychotropic) medication non-adherence will be excluded.

### Selection of studies

We will systematically select studies using predetermined eligibility criteria. The following steps will be used sequentially to select studies for the systematic review and meta-analysis. (1) Studies screening will be based on their “Title”. The titles that are clearly mentioned psychotropic medication non-adherence and associated factors among patients with major psychiatric disorders (schizophrenia, major depressive, and bipolar disorders) will be selected for the subsequent evaluation. (2) Then, the two authors (AS and GT) will independently screen the studies’ abstract section (aim, methods, results and conclusion) to proceed to the next step. In the abstract section, studies which have reported the prevalence of psychotropic medication adherence or medication non-adherence, odds ratio, risk ratio, or relative risk will be included in the final evaluation process. (3) Then, selected studies by abstract screening will be re-assessed for full text (aim, study design, participants selection, tools, results, conclusion and recommendation) by the two authors (AS and GT) independently. (4) With these steps, studies which fulfill the criteria will be included in the systematic review and meta-analysis. However, it is a rare incident to happen, overlapping samples (data) from same source will be evaluated, and the study which has the most representative sample and recently published one will be considered for this systematic review and meta-analysis. Those selected studies will be considered for further appraisal. In case the authors do not agree on any of the studies, studies will be sent to a third person or expert for analysis of quality using same checklist in the view to make final decision. The studies selection process flow diagram will be adapted from the PRISMA guideline [19] (Additional file 3).

### Measurement of outcome and exposure

The main outcome of interest for this systematic review is the level of psychotropic medication non-adherence. Medication non-adherence will be measured either as direct report from studies or indirectly by subtracting adherence report from total observations. Studies reported non-adherence in another way such as medication non-compliance, non-persistence, dropout, discontinuation, missing, and others alternatives will be considered. Moreover, exposure or explanatory variables for the medication non-adherence will be measured using synonymous terms such as determinants, predictors, barriers, associated factors, risk factors, correlates, and influencing factors.

### Quality assurance of the systematic review

Published and unpublished studies will be searched and considered in the systematic review to minimize publication bias. The electronic search engine, manual, and email searching techniques will be applied to have comprehensive search. Eligibility criteria, selection process, and data extraction template will be properly predesigned by the authors to ensure the quality of the systematic review and meta-analysis. The two authors (AS and GT) will evaluate the methodological quality and validity of the studies using the Newcastle-Ottawa Scale (NOS) [21]. The two authors (AS and GT) will perform selection of studies, quality assessment, and data abstractions. Any differences or disagreement will be solved through consensus. Nevertheless, in case of divergent issues, other authors who will not be involved in data extraction, will judge for final decision.

### Data extraction

Two authors (AS and GT) will extract the data from selected studies using data extraction template. Data extraction template will be prepared in Microsoft Excel (2013). Quantitative findings such as number of participant who adhered to the medication (‘labeled as Yes’), non-adhered to the medication (‘labeled as No’) and total participants (n) will be separately presented on the column of the template. In the meantime, qualitative information such as authors' (principal author and date), study area (mainly country), aim of the studies, study design, sample size, sampling procedure, response rate, outcome (non-adherence counts) will be presented on the template. Each studies finding will be presented in a single row. The data extraction template and studies description form is constructed as shown in detail as Additional files 4 and 5.

### Data synthesis

The pooled estimate of medication non-adherence will be computed using Comprehensive Meta-Analysis (CMA) software [22]. Heterogeneity between included studies will be assessed using the I^2^ statistics, where substantial heterogeneity will be assumed if the *I*^2^ value is greater than 75% [23, 24]. However, if studies are heterogeneous (*I*^2^ > 75%), we will narrate the results of each study. Potential publication bias will be checked through visual assessment of the funnel plot [25], Egger’s and Begg’s Test statistics [26]. The associated factors will be qualitatively narrated or synthesized accordingly (individual factors, attitude towards the medication, family or social support related factors, medication or treatment duration factors, health system factors). We will use the random effects model and weighting method [27] to moderate the sample size variation effect on the pooled estimate. In addition, sub-group meta-analysis will be performed for each types of major psychiatric disorders (schizophrenia, major depressive disorders, and bipolar disorders) medication non-adherence. Furthermore, sub-group analysis will be carried out for included studies reported medication non-adherence at different period of assessment.

## Discussion

This systematic review and meta-analysis aims to synthesis available primary research findings on psychotropic medication non-adherence for major psychiatric disorders and associated factors. Even though there is a rough estimate on level of medication non-adherence, it remains a challenging problem in the management of schizophrenia, bipolar and depression disorders. The variation of factors related to non-adherence calls for individually tailored approaches to promote adherence [28, 29]. Psychotropic medication non-adherence contributes to enormous poor health outcomes. This shows substantial work still need to be done to improve medication adherence and treatment effects through evidence based standard care, and informed clinical and public health decision making for patients [28]. Improving adherence in schizophrenia and other psychiatric disorders could have a positive impact on patients and society. Therefore, comprehensive and summarized research evidence is needed to identify key associated factors of the medication non-adherence of patients with major psychiatric disorders. Moreover, research evidence is also required to comprehensively understand the factors of medication non-adherence to address challenges from various points in the health system [29].

Patients given maintenance on antidepressants vary widely in adherence level. As a result, patients’ medication adherence is affected by their perceptions and beliefs about its side effect. Subsequent research has to be carried out to generate concrete evidence on medication non-adherence and associated factors to find appropriate solution for better treatment outcomes of depression patients [30]. Similarly, in the management of bipolar disorders, evidence-based integrated comprehensive intervention is demanded to enhance medication adherence [31]. The promotion to medication adherence should be focused on main factors that affect non-adherence.

The research evidence so far has highlighted that non-adherence is a global challenge in the field of psychiatry, that has linked with poor prognosis of patients on treatment. However, non-adherence is often a hidden issue within consultations of the psychiatrists and other clinicians. Therefore, evidence based interventions should be designed to be enabled patients to overcome perceptual and practical barriers of medication adherence [32]. Patients’ failure to adhere to their medication as prescribed has a major impact on the course of illness and treatment outcomes. Clinicians in psychiatric settings need to elicit information on adherence to their medication to prevent inadequate medications coverage [33].

However, clinicians alone cannot address the issue of medication adherence. It needs a comprehensive approach targeting the major associated factors of the medication non-adherence. However, studies conducted so far do not provide consistent evidence on the level of medication non-adherence and associated factors. Therefore, this systematic review and meta-analysis aims to provide a pooled estimate on the level and associated factors of the medication non-adherence among major psychiatric disorder patients. Our review will generate evidence which will be used to design appropriate interventions to tackle root cause for psychotropic medication non-adherence. Hence, the results of this review and meta-analysis will be helpful for policy-makers, clinicians, and patients to undertake integrated approach for reducing the burden of medication non-adherence for successful major psychiatric disorder treatment outcome.

## Additional files

Additional file 1: PRISMA-P checklist. (DOC 85 kb)

Additional file 2: Search strings. (DOCX 33 kb)

Additional file 3: Figure S1. diagramatic presentation of the selection process of artilces for final systeamtic review. (DOCX 37 kb)

Additional file 4: Table S1. Studies description form. (DOCX 16 kb)

Additional file 5: Data extraction Tamplete. (XLSX 10 kb)

## Abbreviations

CMA: Comprehensive Meta-Analysis
NOS: Newcastle-Ottawa Scale
PRISMA: Preferred Items for Reporting Systematic Review and Meta-analysis
TDR: Tropical Disease Research
US: United States
WHO: World Health Organization
