# Supplementary material for: Psychotropic medication non-adherence and associated factors among adult patients with major psychiatric disorders: a protocol for a systematic review
Source: Syst Rev. 2018 Jan 22;7:10. doi: 10.1186/s13643-018-0676-y (PMC5778728; doi:10.1186/s13643-018-0676-y)
Supplement: Supplementary file 2 — Search strings. (DOCX 33 kb) [file 13643_2018_676_MOESM2_ESM.docx]

**Additional file 2: Search strings**

Psychotropic OR tranquilizer OR sedative OR antidepressant OR mood altering OR antipsychotic AND medication OR treatment OR drug OR remedy AND non-adherence OR adherence OR compliance OR non-compliance OR persistence OR retention AND determinants OR associated factors OR predictors OR influencing factors OR correlates OR barriers OR obstacles OR challenges AND psychiatric disorders OR mental illness OR mental disorders OR major depressive disorders OR schizophrenia OR bipolar disorders OR manic OR depressive illness AND adult patients OR patients aged older than 18 years OR greater than 18 years

| Database | MeSH terms will be used | Sample results |
| --- | --- | --- |
| PubMed | *psychotropic medication OR treatment non-adherence OR non-compliance and Major OR Severe Psychiatric disorders patients* | 78,171 |
|  | *psychotropic medication OR non-adherence OR non-compliance and major psychiatric disorder patients* | 5003 |
|  | *medication non-adherence OR non-compliance among major Psychiatry disorders* | 22,620 |
|  | *(psychotropic medication non-adherence[Title] OR non-compliance[Title] OR adherence[Title] OR compliance among major Psychiatry disorders[Title])* | 23,659 |
|  | *psychotropic medication OR drug OR Treatment non-adherence OR non-compliance OR adherence OR compliance AND adult patient AND major OR severe Psychiatry disorders* | 70,248 |
|  | *(((psychotropic medication OR drug OR Treatment non-adherence OR non-compliance OR adherence OR compliance A AND)) AND adult patient) AND (major OR severe psychiatry disorders OR illness OR disease)* | [329960](https://www.ncbi.nlm.nih.gov/pubmed/?cmd=HistorySearch&querykey=7) |
|  | *((((psychotropic medication non-adherence[Title] OR non-compliance[Title] OR adherence[Title] OR compliance among major Psychiatry disorders[Title]))) AND (medication non-adherence OR non-compliance among major Psychiatry disorders)) AND (associated factors OR determinants OR predictors OR risk factors OR barriers)* | 3169 |
|  | *(((psychotropic medication non-adherence[Title] OR non-compliance[Title] OR adherence[Title] OR compliance)) AND (schizophrenia OR Depression OR bipolar)* | 1017 |
|  | *((psychotropic medication non-adherence[Title] OR non-compliance[Title] OR adherence[Title] OR compliance)) AND (schizophrenia OR depression OR bipolar)* | 8882 |
|  | *(((((psychotropic medication non-adherence[Title] OR non-compliance[Title] OR adherence[Title] OR compliance)) AND (schizophrenia OR depression OR bipolar))) AND (Associated factors OR determinants OR predictors OR barriers)) AND Adult patient* | 1304 |

(Psychotropic OR tranquilizer OR sedative OR antidepressant OR mood altering OR antipsychotic) AND (medication OR treatment OR drug OR remedy) AND (non-adherence OR, adherence OR compliance OR non-compliance OR persistence OR) AND (determinants OR associated factors OR predictors OR influencing factors OR correlates OR barriers) AND (psychiatric disorders OR mental illness OR mental disorders OR major depressive disorders OR schizophrenia OR bipolar disorders OR manic depressive illness) AND (adult patients OR patients aged older than 18 years OR greater than 18 years)

PubMed=625

Medline: ((((((((((Psychotropic or tranquilizer or sedative or antidepressant or mood altering or antipsychotic) and medication or treatment or drug or remedy and non-adherence) or adherence or compliance or non-compliance or persistence and determinants) or associated factors or predictors or influencing factors or correlates or barriers) and psychiatric disorders) or mental illness or mental disorders or major depressive disorders or schizophrenia or bipolar disorders or manic depressive illness) and adult patients) or patients aged older than 18 years or greater than 18 years) .m_titl. ===38

CINAHL=55

| S1 | psychotropic medication OR tranquilizer OR sedative drugs OR antidepressants OR mind altering drugs OR antipsychotic medication | **Search modes** - Boolean/Phrase | [**View Results**](javascript:__doPostBack('ctl00$ctl00$FindField$FindField$historyControl$HistoryRepeater$ctl00$linkResults','')) (15,449) |
| --- | --- | --- | --- |

| S2 | medication adherence OR treatment OR drug therapy OR remedy | **Search modes** - Boolean/Phrase | [**View Results**](javascript:__doPostBack('ctl00$ctl00$FindField$FindField$historyControl$HistoryRepeater$ctl00$linkResults','')) (980,271) |
| --- | --- | --- | --- |

|  | S3 | ( nonadherence or noncompliance ) OR adhrence OR compliance OR persistence OR non-compliance OR retention | **Search modes** - Boolean/Phrase | [**View Results**](javascript:__doPostBack('ctl00$ctl00$FindField$FindField$historyControl$HistoryRepeater$ctl00$linkResults','')) (99,490) |
| --- | --- | --- | --- | --- |

| S4 | determinants OR associated factors OR predictors OR correlates OR influencing factors OR ( barriers or obstacles or challenges ) | **Search modes** - Boolean/Phrase | [**View Results**](javascript:__doPostBack('ctl00$ctl00$FindField$FindField$historyControl$HistoryRepeater$ctl00$linkResults','')) (368,065) |
| --- | --- | --- | --- |

| S5 | psychiatric disorders OR mental illness OR mental disorders OR major depressive disorder OR schizophrenia OR bipolar disorder OR mania OR depressive disorder | **Search modes** - Boolean/Phrase | [**View Results**](javascript:__doPostBack('ctl00$ctl00$FindField$FindField$historyControl$HistoryRepeater$ctl00$linkResults','')) (102,187) |
| --- | --- | --- | --- |

| S6 | ( adult patients or adults ) OR patients older than 18 years OR greater than 18 years | **Search modes** - Boolean/Phrase | [**View Results**](javascript:__doPostBack('ctl00$ctl00$FindField$FindField$historyControl$HistoryRepeater$ctl00$linkResults','')) (1,009,979) |
| --- | --- | --- | --- |

|  | S7 | S1 AND S2 AND S3 AND S4 AND S5 AND S6 | **Search modes** - Boolean/Phrase | [**View Results**](javascript:__doPostBack('ctl00$ctl00$FindField$FindField$historyControl$HistoryRepeater$ctl00$linkResults','')) (55) |
| --- | --- | --- | --- | --- |

[Accessibility Information and Tips](javascript:openWideTip('http://support.ebsco.com.ezproxy.newcastle.edu.au/help/?int=ehost&lang=en&feature_id=access&TOC_ID=Always&SI=0&BU=0&GU=1&PS=0&ver=&dbs=ccm'))Revised Date: 07/2015

**Print Search History**

| 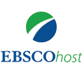 | Wednesday, August 23, 2017 3:45:50 AM |
| --- | --- |

| **#** | **Query** | **Limiters/Expanders** | **Last Run Via** | **Results** |
| --- | --- | --- | --- | --- |
| S8 | S1 AND S2 AND S3 AND S4 AND S5 AND S6 | Limiters - Full Text  Search modes - Boolean/Phrase | Interface - EBSCOhost Research Databases  Search Screen - Advanced Search  Database - CINAHL Complete | 51 |
| S7 | S1 AND S2 AND S3 AND S4 AND S5 AND S6 | Search modes - Boolean/Phrase | Interface - EBSCOhost Research Databases  Search Screen - Advanced Search  Database - CINAHL Complete | 55 |
| S6 | ( adult patients or adults ) OR patients older than 18 years OR greater than 18 years | Search modes - Boolean/Phrase | Interface - EBSCOhost Research Databases  Search Screen - Advanced Search  Database - CINAHL Complete | 1,009,979 |
| S5 | psychiatric disorders OR mental illness OR mental disorders OR major depressive disorder OR schizophrenia OR bipolar disorder OR mania OR depressive disorder | Search modes - Boolean/Phrase | Interface - EBSCOhost Research Databases  Search Screen - Advanced Search  Database - CINAHL Complete | 102,187 |
| S4 | determinants OR associated factors OR predictors OR correlates OR influencing factors OR ( barriers or obstacles or challenges ) | Search modes - Boolean/Phrase | Interface - EBSCOhost Research Databases  Search Screen - Advanced Search  Database - CINAHL Complete | 368,065 |
| S3 | ( nonadherence or noncompliance ) OR adhrence OR compliance OR persistence OR non-compliance OR retention | Search modes - Boolean/Phrase | Interface - EBSCOhost Research Databases  Search Screen - Advanced Search  Database - CINAHL Complete | 99,490 |
| S2 | medication adherence OR treatment OR drug therapy OR remedy | Search modes - Boolean/Phrase | Interface - EBSCOhost Research Databases  Search Screen - Advanced Search  Database - CINAHL Complete | 980,271 |
| S1 | psychotropic medication OR tranquilizer OR sedative drugs OR antidepressants OR mind altering drugs OR antipsychotic medication | Search modes - Boolean/Phrase | Interface - EBSCOhost Research Databases  Search Screen - Advanced Search  Database - CINAHL Complete | 15,449 |

Medline: 1222

(((((((((((Psychotropic or tranquilizer or sedative or antidepressant or mood altering or antipsychotic) and medication) or treatment or drug or remedy) and non-adherence) or adherence or compliance or non-compliance or persistence or retention) and determinants) or associated factors or predictors or influencing factors or correlates or barriers or obstacles or challenges) and psychiatric disorders) or mental illness or mental disorders or major depressive disorders or schizophrenia or bipolar disorders or manic or depressive illness) and adult patients) or patients aged older than 18 years or greater than 18 years).mp. [mp=title, abstract, original title, name of substance word, subject heading word, keyword heading word, protocol supplementary concept word, rare disease supplementary concept word, unique identifier, synonyms]

### Web science = 12,993,551

(((((((((((((((((((((((((((((**TOPIC:**(Psychotropic) *OR* **MeSH HEADING:exp:** (Psychotropic Drugs)) *OR* (**TOPIC:** (tranquilizer) *OR* (**MeSH HEADING:exp:** ((Substance-Related Disorders)) *AND* **MeSH HEADING:exp:** ((Conduct Disorder)))**MeSH HEADING:exp:**((Hypnotics and Sedatives)) *OR***MeSH HEADING:exp:** (Tranquilizing Agents))) *OR* (**TOPIC:** (sedative) *OR***MeSH HEADING:exp:** ((Hypnotics and Sedatives)) *OR* (**MeSH HEADING:exp:** ((Hypnotics and Sedatives)) *AND* **MeSH HEADING:exp:** ((Psychotropic Drugs))))) *OR* (**TOPIC:**(antidepressant) *OR* **MeSH HEADING:exp:** (Antidepressive Agents))) *OR* ((**TOPIC:** (mood) *OR***MeSH HEADING:exp:** (Biological Processes)) *AND* **TOPIC:** (altering))) *OR* ((**TOPIC:** (antipsychotic) *OR***MeSH HEADING:exp:** (Antipsychotic Agents)) *AND* (**TOPIC:** (medication) *OR* **MeSH HEADING:exp:** (Nursing Care) *OR* **MeSH HEADING:exp:**(Pharmaceutical Preparations) *OR***MeSH HEADING:exp:**(Pharmaceutical Preparations) *OR***MeSH HEADING:exp:** (Nursing Care) *OR* **MeSH HEADING:exp:**(Pharmaceutical Preparations)))) *OR*(**TOPIC:** (treatment) *OR* (**MeSH HEADING:exp:** ((Organization and Administration)) *OR* **MeSH HEADING:exp:**((Therapeutics)))**MeSH HEADINGS:exp:** (/therapy) *OR* **MeSH HEADING:exp:** (Therapeutics) *OR***MeSH HEADING:exp:**(Therapeutics))) *OR* (**TOPIC:** (drug) *OR* **MeSH HEADING:exp:**(Pharmaceutical Preparations) *OR***MeSH HEADING:exp:** (Dental Care) *OR* **MeSH HEADING:exp:**(Pharmaceutical Preparations) *OR***MeSH HEADING:exp:** (Anti-Infective Agents))) *OR* **TOPIC:** (remedy AND non-adherence)) *OR* **TOPIC:**(adherence)) *OR* (**TOPIC:**(compliance) *OR* **MeSH HEADING:exp:** (Compliance) *OR***MeSH HEADING:exp:** (Compliance) *OR* **MeSH HEADING:exp:** (Patient Compliance))) *OR* (**TOPIC:** (non-compliance) *OR* **MeSH HEADING:exp:** (Patient Compliance))) *OR* (**TOPIC:**(persistence) *OR* **MeSH HEADING:exp:** (Attention))) *OR*((**TOPIC:** (retention) *OR* **MeSH HEADING:exp:** (Biological Processes) *OR* **MeSH HEADING:exp:** (Retention (Psychology)) *OR* **MeSH HEADING:exp:** (Urinary Retention)) *AND* **TOPIC:** (determinants))) *OR***TOPIC:** (associated factors)) *OR***TOPIC:** (predictors)) *OR* **TOPIC:**(influencing factors)) *OR* **TOPIC:**(correlates)) *OR* **TOPIC:** (barriers)) *OR* **TOPIC:** (obstacles)) *OR* (**TOPIC:**(challenges) *AND* (**TOPIC:**(psychiatric disorders) *OR* **MeSH HEADING:exp:** (Mental Disorders)))) *OR* (**TOPIC:** (mental illness) *OR***MeSH HEADING:exp:** (Mental Disorders))) *OR* (**TOPIC:** (mental disorders) *OR* **MeSH HEADING:exp:**(Mental Disorders) *OR* **MeSH HEADING:exp:** (Psychotic Disorders))) *OR* (**TOPIC:** (major depressive disorders) *OR* **MeSH HEADING:exp:** (Depressive Disorder, Major) *OR* **MeSH HEADING:exp:**(Depressive Disorder))) *OR* (**TOPIC:**(schizophrenia) *OR* **MeSH HEADING:exp:** (Schizophrenia))) *OR*(**TOPIC:** (bipolar disorders) *OR* **MeSH HEADING:exp:** (Bipolar Disorder))) *OR* (**TOPIC:** (manic) *OR* **MeSH HEADING:exp:** (Bipolar Disorder))) *OR* ((**TOPIC:** (depressive illness) *OR***MeSH HEADING:exp:** (Depressive Disorder)) *AND* ((**TOPIC:** (adult) *OR***MeSH HEADING:exp:** (Adult)) *AND*(**TOPIC:** (patients) *OR* **MeSH HEADING:exp:** (Patients))))) *OR*((**TOPIC:** (patients) *OR* **MeSH HEADING:exp:** (Patients)) *AND*(**TOPIC:** (aged) *OR* **MeSH HEADING:exp:** (Aged) *OR* **MeSH HEADING:exp:** (Aging) *OR* **MeSH HEADING:exp:** (Aged) *OR* **MeSH HEADING:exp:** (Glycosylation End Products, Advanced) *OR* **MeSH HEADING:exp:** (Aged))**TOPIC:**(older) *OR* **TOPIC:** (than) *OR* (**TOPIC:**(18) *OR* **MeSH HEADING:exp:**(Occipital Lobe) *OR* **MeSH HEADING:exp:** (Molar) *OR* **MeSH HEADING:exp:** (Molar))(**TOPIC:**(years) *OR* **MeSH HEADING:exp:**(Periodicity) *OR* **MeSH HEADINGS:exp:** (/pathology)))) *OR*(**TOPIC:** (greater) *AND* **TOPIC:** (than) *AND* (**TOPIC:** (18) *OR* **MeSH HEADING:exp:** (Occipital Lobe) *OR***MeSH HEADING:exp:** (Molar) *OR***MeSH HEADING:exp:** (Molar))(**TOPIC:** (years) *OR* **MeSH HEADING:exp:** (Periodicity) *OR***MeSH HEADINGS:exp:** (/pathology))

### Web science: Results: 963

*(from MEDLINE)*

**You searched for:**(((((((((((((((((((((((((((((**TOPIC:** (Psychotropic) *OR* **MeSH HEADING:exp:** (Psychotropic Drugs)) *OR* (**TOPIC:** (tranquilizer) *OR* (**MeSH HEADING:exp:** ((Substance-Related Disorders)) *AND* **MeSH HEADING:exp:** ((Conduct Disorder)))**MeSH HEADING:exp:** ((Hypnotics and Sedatives)) *OR* **MeSH HEADING:exp:** (Tranquilizing Agents))) *OR* (**TOPIC:** (sedative) *OR* **MeSH HEADING:exp:** ((Hypnotics and Sedatives)) *OR* (**MeSH HEADING:exp:** ((Hypnotics and Sedatives)) *AND* **MeSH HEADING:exp:** ((Psychotropic Drugs))))) *OR*(**TOPIC:** (antidepressant) *OR* **MeSH HEADING:exp:** (Antidepressive Agents))) *OR* ((**TOPIC:** (mood) *OR* **MeSH HEADING:exp:** (Biological Processes)) *AND* **TOPIC:** (altering))) *OR* ((**TOPIC:**(antipsychotic) *OR* **MeSH HEADING:exp:** (Antipsychotic Agents)) *AND* (**TOPIC:** (medication) *OR* **MeSH HEADING:exp:** (Nursing Care) *OR* **MeSH HEADING:exp:** (Pharmaceutical Preparations) *OR* **MeSH HEADING:exp:** (Pharmaceutical Preparations) *OR* **MeSH HEADING:exp:** (Nursing Care) *OR* **MeSH HEADING:exp:** (Pharmaceutical Preparations)))) *OR* (**TOPIC:** (treatment) *OR* (**MeSH HEADING:exp:** ((Organization and Administration)) *OR* **MeSH HEADING:exp:** ((Therapeutics)))**MeSH HEADINGS:exp:** (/therapy) *OR***MeSH HEADING:exp:** (Therapeutics) *OR* **MeSH HEADING:exp:** (Therapeutics))) *OR* (**TOPIC:** (drug) *OR* **MeSH HEADING:exp:** (Pharmaceutical Preparations) *OR* **MeSH HEADING:exp:** (Dental Care) *OR* **MeSH HEADING:exp:**(Pharmaceutical Preparations) *OR* **MeSH HEADING:exp:** (Anti-Infective Agents))) *OR* **TOPIC:** (remedy AND non-adherence)) *OR* **TOPIC:** (adherence)) *OR* (**TOPIC:** (compliance) *OR* **MeSH HEADING:exp:** (Compliance) *OR* **MeSH HEADING:exp:** (Compliance) *OR***MeSH HEADING:exp:** (Patient Compliance))) *OR* (**TOPIC:** (non-compliance) *OR* **MeSH HEADING:exp:** (Patient Compliance))) *OR* (**TOPIC:** (persistence) *OR* **MeSH HEADING:exp:** (Attention))) *OR* ((**TOPIC:** (retention) *OR* **MeSH HEADING:exp:** (Biological Processes) *OR* **MeSH HEADING:exp:** (Retention (Psychology)) *OR* **MeSH HEADING:exp:** (Urinary Retention)) *AND* **TOPIC:** (determinants))) *OR* **TOPIC:** (associated factors)) *OR* **TOPIC:** (predictors)) *OR* **TOPIC:** (influencing factors)) *OR* **TOPIC:** (correlates)) *OR* **TOPIC:**(barriers)) *OR* **TOPIC:** (obstacles)) *OR* (**TOPIC:** (challenges) *AND* (**TOPIC:**(psychiatric disorders) *OR* **MeSH HEADING:exp:** (Mental Disorders)))) *OR*(**TOPIC:** (mental illness) *OR* **MeSH HEADING:exp:** (Mental Disorders))) *OR* (**TOPIC:** (mental disorders) *OR* **MeSH HEADING:exp:** (Mental Disorders) *OR* **MeSH HEADING:exp:** (Psychotic Disorders))) *OR* (**TOPIC:** (major depressive disorders) *OR* **MeSH HEADING:exp:** (Depressive Disorder, Major) *OR* **MeSH HEADING:exp:** (Depressive Disorder))) *OR* (**TOPIC:** (schizophrenia) *OR* **MeSH HEADING:exp:** (Schizophrenia))) *OR* (**TOPIC:** (bipolar disorders) *OR* **MeSH HEADING:exp:** (Bipolar Disorder))) *OR* (**TOPIC:** (manic) *OR* **MeSH HEADING:exp:** (Bipolar Disorder))) *OR* ((**TOPIC:** (depressive illness) *OR* **MeSH HEADING:exp:** (Depressive Disorder)) *AND* ((**TOPIC:** (adult) *OR* **MeSH HEADING:exp:** (Adult)) *AND* (**TOPIC:** (patients) *OR* **MeSH HEADING:exp:** (Patients))))) *OR* ((**TOPIC:**(patients) *OR* **MeSH HEADING:exp:**(Patients)) *AND* (**TOPIC:** (aged) *OR* **MeSH HEADING:exp:** (Aged) *OR* **MeSH HEADING:exp:** (Aging) *OR* **MeSH HEADING:exp:** (Aged) *OR* **MeSH HEADING:exp:** (Glycosylation End Products, Advanced) *OR* **MeSH HEADING:exp:** (Aged))**TOPIC:** (older) *OR* **TOPIC:** (than) *OR* (**TOPIC:** (18) *OR* **MeSH HEADING:exp:** (Occipital Lobe) *OR***MeSH HEADING:exp:** (Molar) *OR* **MeSH HEADING:exp:** (Molar))(**TOPIC:**(years) *OR* **MeSH HEADING:exp:** (Periodicity) *OR* **MeSH HEADINGS:exp:**(/pathology)))) *OR* (**TOPIC:** (greater) *AND* **TOPIC:** (than) *AND* (**TOPIC:** (18) *OR* **MeSH HEADING:exp:** (Occipital Lobe) *OR* **MeSH HEADING:exp:**(Molar) *OR* **MeSH HEADING:exp:** (Molar))(**TOPIC:** (years) *OR* **MeSH HEADING:exp:** (Periodicity) *OR* **MeSH HEADINGS:exp:** (/pathology))) [**...More**](javascript:void(0))

EMBASE =50

((((((((((Psychotropic or tranquilizer or sedative or antidepressant or mood altering or antipsychotic) and medication) or treatment or drug or remedy) and non-adherence) or adherence or compliance or non-compliance or persistence or retention) and determinants) or associated factors or predictors or influencing factors or correlates or barriers or obstacles or challenges) and psychiatric disorders) or mental illness or mental disorders or major depressive disorders or schizophrenia or bipolar disorders or manic or depressive illness) and adult patients) or patients aged older than 18 years or greater than 18 years).m_titl.
