# Supplementary material for: Psychotropic medication non-adherence and associated factors among adult patients with major psychiatric disorders: a protocol for a systematic review
Source: Syst Rev. 2018 Jan 22;7:10. doi: 10.1186/s13643-018-0676-y (PMC5778728; doi:10.1186/s13643-018-0676-y)
Supplement: Supplementary file 3 — diagramatic presentation of the selection process of artilces for final systeamtic review. (DOCX 37 kb) [file 13643_2018_676_MOESM3_ESM.docx]

Additional file 2

| Additional studies collected using other relevant sources (email request, thesis/dissertation and other data sharing mechanisms)  Potentially relevant studies and publications obtain through database searching activities and email (n)  Number of studies after removal of duplications  Papers excluded on the basis of title (n)  Number of studies included based on abstract (n)  Papers excluded based on reviewing the abstract parts (n)  Papers eligible for the whole body review for eligibility to be included to the final review (n)  Exclude from the review after assessing the full text of the paper. The papers did not clearly reported prevalence and associated factors (n)  Relevant information to be systematically review and extracted (n)  Number of studies used in the qualitative synthesis (systematic review)  Number of studies used for quantitative meta-analysis |
| --- |
| Figure S1: diagramatic presentation of the selection process of artilces for final systeamtic review |
