# Supplementary material for: Psychotropic medication non-adherence and associated factors among adult patients with major psychiatric disorders: a protocol for a systematic review
Source: Syst Rev. 2018 Jan 22;7:10. doi: 10.1186/s13643-018-0676-y (PMC5778728; doi:10.1186/s13643-018-0676-y)
Supplement: Supplementary file 4 — Studies description form. (DOCX 16 kb) [file 13643_2018_676_MOESM4_ESM.docx]

Table S1: Studies description form

| Author, country | study aim | design | Population | Sampling procedure | Sample size | Response | Main findings | Key conclusion and recommendation |
| --- | --- | --- | --- | --- | --- | --- | --- | --- |
| Abdu Wakawa Ibrahim et al, 2015  Nigeria | To determine the socio-demographic and clinical predictors of sub-optimal adherence among the subjects. | Cross-sectional | Adults Patients (>18 years) with Schizophrenia, depression, | SRS | 390 | 94.8% | Medication non-adherence was 55.7%. Seeking for traditional treatment (OR, 6.5, 95% CI: 3.8, 11.3), male (OR,3.3, 95% CI: 1.9, 5.7), low levels of insight (OR, 1.8 95% CI:1.2, 2.5), and low social support levels (OR, 1.5, 95% CI: 1.1, 2.1) were predictors | Psycho-educational programmes on adherence and the active involvement of the relations and significant others in the management of patients |
| Eticha,T. et al, 2015.Ethiopia | To investigate factors associated with medication adherence among patients with schizophrenia | Cross sectional | patients with schizophrenia (aged 18 to 65 years) | Consecutive schizophrenic patients | 393 | 97.5 | Non-adherence was 26.5%. Positive attitude (AOR, 1.4, 95% CI:1.3,1.6),side effects (AOR,1.0, 95% CI:0.9, 1.0), awareness of illness (AOR, 1.4, 95% CI:1.1,1.9) and reliable symptoms (AOR,1.6, 95% CI:1.2,2.1). Khat (AOR, 0.2, 95% CI: 0.1, 0.7), illiterate (AOR, 0.13, 95% CI:0.03, 0.5) and older age (AOR, 0.03, 95% CI:0.1,0.2) were associated factors. | Schizophrenia patients were highly non-adherence to their medication. Intervention strategies focused on educating the patients to better understand the illness, medications and their potential side effects might be useful in improving adherence. |
| Banerjee S, 2012, India (Unpublished) | Assessing the correlates of non-adherence to unipolar patients | Cross-sectional | Psychiatric with depression | Purposive sampling | 239 | 97.2% | Medication non-adherence was 66.9%. Women (OR 2.7; 95% CI 1.0, 7.1), consume extra pills than the recommended number (OR 2.8; 95% CI 1.1, 7.3) and had a considerably lower internal locus of control (OR 4.5; 95% CI 2.4 - 8.3) were predictors | Interventions focusing on individuals and intersectoral systems oriented approach to improve adherence is needed for treatment strategies to be effective |
| Shreya Dave, 2012. UK | To assess the patterns, incidence and predictors of therapy discontinuation among MDD patients | Retrospective cohort | patients with MDD between 2006-2008 | Random sampling | 13,927 | 91.2% | Discontinuation of antidepressant therapy over follow-up was 80.1. 42% of patients had discontinued at six month and 33% had ceased therapy altogether. Lower dis- continuation in the first six months after initiation was associated with higher age, weight gain and comorbid irritable bowel syndrome. | Significant risk of discontinuation of antidepressant therapy in the 6 months after initiation of treatment for MDD. Awareness by the physician to ensure implementation of optimal treatment and minimization non-compliance |
|  |  |  |  |  |  |  |  |  |
|  |  |  |  |  |  |  |  |  |
|  |  |  |  |  |  |  |  |  |
|  |  |  |  |  |  |  |  |  |
|  |  |  |  |  |  |  |  |  |
|  |  |  |  |  |  |  |  |  |
|  |  |  |  |  |  |  |  |  |
|  |  |  |  |  |  |  |  |  |
|  |  |  |  |  |  |  |  |  |
|  |  |  |  |  |  |  |  |  |
|  |  |  |  |  |  |  |  |  |
|  |  |  |  |  |  |  |  |  |
